# Supplementary material for: The influence of health awareness on university students’ healthy lifestyles: The chain mediating role of self-esteem and social support
Source: PLoS One. 2024 Oct 10;19(10):e0311886. doi: 10.1371/journal.pone.0311886 (PMC11466429; doi:10.1371/journal.pone.0311886)
Supplement: Attachment — Submitted filename: plosreview1.pdf [file pone.0311886.s001.pdf]

The Influence of Health Awareness on University Students' Healthy Lifestyles: The Chain Mediating Role of Self-esteem and Social Support

Strengths:

Sequential and Well-Organized: The paper's information is presented sequentially and is well-organized, making it easy to follow.

Areas for Improvement:

Clarify Research Objective: The research objective should be explicitly stated at the beginning of the paper.

Problem Statement: The problem statement needs more clarity. How was the hypothesis developed? Was it based on theoretical foundations or identified through a literature gap?

Mediator Role: Provide a detailed explanation of the mediator's role and its function within the context of this research.

Sample Selection Process: Elaborate on how the sample was selected.

Online Survey Process and Limitations:

Detail the process by which participants voluntarily participated and submitted their questionnaires through an online survey.

Discuss any limitations associated with using an online survey and how these were mitigated.

Instrument Translation: If the instrument was translated, describe the translation process and how the translated instrument reached the sample.

Recommendations:

Literature Review Section: Create a separate literature review section instead of including it in the introduction.

Discussion, Limitations, and Future Research: These sections should be thoroughly explained and clearly outlined.
