# Supplementary material for: The influence of health awareness on university students’ healthy lifestyles: The chain mediating role of self-esteem and social support
Source: PLoS One. 2024 Oct 10;19(10):e0311886. doi: 10.1371/journal.pone.0311886 (PMC11466429; doi:10.1371/journal.pone.0311886)
Supplement: Attachment — Submitted filename: PONE-D-24-27865_REVIEWED.docx [file pone.0311886.s002.docx]

PLOS ONE

# The Influence of Health Awareness on University Students' Healthy Lifestyles: The Chain Mediating Role of Self-esteem and Social Support

--Manuscript Draft--

| Manuscript Number: | PONE-D-24-27865 |
| --- | --- |
| Article Type: | Research Article |
| Full Title: | The Influence of Health Awareness on University Students' Healthy Lifestyles: The Chain Mediating Role of Self-esteem and Social Support |
| Short Title: | The Influence of Health Awareness on University Students' Healthy Lifestyles |
| Corresponding Author: | Zeqing Zhang  Universiti Putra Malaysia Serdang, Selangor MALAYSIA |
| Keywords: | health awareness; healthy lifestyles; self-esteem; social support |
| Abstract: | This study aims to explore the relationship between health awareness (HA) and healthy lifestyles (HL), as well as the mediating roles of self-esteem (SE) and social support (SS). Based on social cognitive theory, this study initially constructed a theoretical model to examine the impact of health awareness on healthy lifestyles among university students. Self-esteem and social support were introduced as mediating variables to further develop a chain mediation model. A total of 1169 participants (554 males and 615 females) completed an online questionnaire survey. In this study, participants filled out the Health Awareness Scale (HAS), the Diet and Healthy Lifestyle Scale (DEVS), the Rosenberg Self-esteem Scale (RSES), and the Oslo Social Support Scale (OSSS-3). The results showed that high levels of health awareness positively influenced self-esteem, which in turn positively affected social support, ultimately leading to a positive impact on healthy lifestyles. Specifically, self- esteem and social support played significant mediating roles between health awareness and healthy lifestyles. Overall, the findings reveal the pathways through which health awareness affects healthy lifestyles, highlighting important implications for designing effective interventions to promote healthy lifestyles. |
| Order of Authors: | Zeqing Zhang |
|  | Haslinda Abdullah |
|  | Akmar Hayati Ahmad Ghazali |
|  | Jeffrey Lawrence D’Silva |
|  | Ismi Arif Ismail |
|  | Zerui Huang |
| Additional Information: |  |
| Question | Response |
| Financial Disclosure  Enter a financial disclosure statement that describes the sources of funding for the work included in this submission. Review the [submission guidelines](http://journals.plos.org/plosone/s/submission-guidelines#loc-financial-disclosure-statement) for detailed requirements. View published research articles from [*PLOS ONE*](https://journals.plos.org/plosone/) for specific examples.  This statement is required for submission | The author(s) received no specific funding for this work. |

*Powered by Editorial Manager® and ProduXion Manager® from Aries Systems Corporation*

| and will appear in the published article if the submission is accepted. Please make sure it is accurate. |  |
| --- | --- |
| Funded studies  Enter a statement with the following details:   - Initials of the authors who received each award - Grant numbers awarded to each author - The full name of each funder - URL of each funder website - Did the sponsors or funders play any role in the study design, data collection and analysis, decision to publish, or preparation of the manuscript? |  |
| Did you receive funding for this work? |  |
| Competing Interests  Use the instructions below to enter a competing interest statement for this submission. On behalf of all authors, disclose any [competing interests](http://journals.plos.org/plosone/s/competing-interests) that could be perceived to bias this work—acknowledging all financial support and any other relevant financial or non- financial competing interests.  This statement is required for submission and will appear in the published article if the submission is accepted. Please make sure it is accurate and that any funding sources listed in your Funding Information later in the submission form are also declared in your Financial Disclosure statement.  View published research articles from  [*PLOS ONE*](http://journals.plos.org/plosone/) for specific examples. | The authors have declared that no competing interests exist. |

*Powered by Editorial Manager® and ProduXion Manager® from Aries Systems Corporation*

| NO authors have competing interests  Enter: *The authors have declared that no competing interests exist.*  Authors with competing interests  Enter competing interest details beginning with this statement:  *I have read the journal's policy and the authors of this manuscript have the following competing interests: [insert competing interests here]* |  |
| --- | --- |
| * typeset |  |
| Ethics Statement  Enter an ethics statement for this submission. This statement is required if the study involved:   - Human participants - Human specimens or tissue - Vertebrate animals or cephalopods - Vertebrate embryos or tissues - Field research   Write "N/A" if the submission does not require an ethics statement.  General guidance is provided below. Consult the [submission guidelines](http://journals.plos.org/plosone/s/submission-guidelines#loc-guidelines-for-specific-study-types) for detailed instructions. Make sure that all information entered here is included in the Methods section of the manuscript. | This study involved human participants. The research was approved by the Ethics Committee of Guangdong Business and Technology University on February 5, 2024, with the approval number 2024GS006. All participants provided written informed consent before taking part in the study. The consent process ensured that participants fully understood the purpose, process, and potential impacts of the study. The study was conducted anonymously to protect participants' privacy and confidentiality. |

*Powered by Editorial Manager® and ProduXion Manager® from Aries Systems Corporation*

| Format for specific study types  Human Subject Research (involving human participants and/or tissue)   - Give the name of the institutional review board or ethics committee that approved the study - Include the approval number and/or a statement indicating approval of this research - Indicate the form of consent obtained (written/oral) or the reason that consent was not obtained (e.g. the data were analyzed anonymously)   Animal Research (involving vertebrate animals, embryos or tissues)   - Provide the name of the Institutional Animal Care and Use Committee (IACUC) or other relevant ethics board that reviewed the study protocol, and indicate whether they approved this research or granted a formal waiver of ethical approval - Include an approval number if one was obtained - If the study involved *non-human primates*, add *additional details* about animal welfare and steps taken to ameliorate suffering - If anesthesia, euthanasia, or any kind of animal sacrifice is part of the study, include briefly which substances and/or methods were applied   Field Research  Include the following details if this study involves the collection of plant, animal, or other materials from a natural setting:   - Field permit number - Name of the institution or relevant body that granted permission |  |
| --- | --- |
|  |  |
| Data Availability  Authors are required to make all data underlying the findings described fully available, without restriction, and from the time of publication. PLOS allows rare exceptions to address legal and ethical concerns. See the [PLOS Data Policy](http://journals.plos.org/plosone/s/data-availability) and [FAQ](http://journals.plos.org/plosone/s/data-availability#loc-faqs-for-data-policy) for detailed information. | Yes - all data are fully available without restriction |

*Powered by Editorial Manager® and ProduXion Manager® from Aries Systems Corporation*

| A Data Availability Statement describing where the data can be found is required at submission. Your answers to this question constitute the Data Availability Statement and will be published in the article, if accepted. |  |
| --- | --- |
| Important: Stating ‘data available on request from the author’ is not sufficient. If your data are only available upon request, select ‘No’ for the first question and explain your exceptional situation in the text box. |  |
| Do the authors confirm that all data underlying the findings described in their manuscript are fully available without restriction? |  |
| Describe where the data may be found in full sentences. If you are copying our sample text, replace any instances of XXX with the appropriate details.   - If the data are held or will be held in a public repository, include URLs, accession numbers or DOIs. If this information will only be available after acceptance, indicate this by ticking the box below. For example: *All XXX files are available from the XXX database (accession number(s) XXX, XXX.*). - If the data are all contained within the manuscript and/or Supporting Information files, enter the following: *All relevant data are within the manuscript and its Supporting Information files.* - If neither of these applies but you are able to provide details of access elsewhere, with or without limitations, please do so. For example:   *Data cannot be shared publicly because of [XXX]. Data are available from the XXX Institutional Data Access / Ethics Committee (contact via XXX) for researchers who meet the criteria for access to confidential data.*  *The data underlying the results presented in the study are available from (include the name of the third party* | All relevant data are within the manuscript and its Supporting Information files. |

*Powered by Editorial Manager® and ProduXion Manager® from Aries Systems Corporation*

| *and contact information or URL).*   - This text is appropriate if the data are owned by a third party and authors do not have permission to share the data.   * typeset |  |
| --- | --- |
| Additional data availability information: |  |

*Powered by Editorial Manager® and ProduXion Manager® from Aries Systems Corporation*

Manuscript	[Click here to access/download;Manuscript;Manuscript.docx](https://www2.cloud.editorialmanager.com/pone/download.aspx?id=36841540&guid=477a1e1d-09df-47e9-8107-16916fbe4195&scheme=1)[<https://www2.cloud.editorialmanager.com/pone/download.aspx?id=36841540&guid=477a1e1d-09df-47e9-8107-16916fbe4195&scheme=1>](https://www2.cloud.editorialmanager.com/pone/download.aspx?id=36841540&guid=477a1e1d-09df-47e9-8107-16916fbe4195&scheme=1)

#### The Influence of Health Awareness on University Students' Healthy Lifestyles: The Chain Mediating Role of Self-esteem and Social Support

Zeqing Zhang^1,2^, Haslinda Abdullah^1^, Akmar Hayati Ahmad Ghazali^3^, Jeffrey Lawrence D’Silva^1^, Ismi Arif Ismail^4^, Zerui Huang^1,2^

^1^Institute for Social Science Studies, Universiti Putra Malaysia, 43400 UPM, Serdang, Selangor, Malaysia;

^2^School of Culture and Communication, Guangdong Business and Technology University, Zhaoqing, People’s Republic of China;

^3^Faculty of Modern Languages and Communication, Universiti Putra Malaysia, 43400 UPM, Serdang, Selangor, Malaysia;

^4^Department of Professional Development and Continuing Education, Faculty of Educational Studies, Universiti Putra Malaysia, 43400 UPM, Serdang, Selangor, Malaysia.

**Correspondence:** Zeqing Zhang, Institute for Social Science Studies, Universiti Putra Malaysia, 43400 UPM, Serdang, Selangor, Malaysia, Tel +86 18735755857, Email zhangzqluna@foxmail.com; Zerui Huang, School of Culture and Communication, Guangdong Business and Technology University, Duanzhou District, Zhaoqing, Guangdong Province, 526020, People’s Republic of China, Tel +86 15013230239, Email [huangzrio@foxmail.com](mailto:huangzrio@foxmail.com)

#### ABSTRACT

This study aims to explore the relationship between health awareness (HA) and healthy lifestyles (HL), as well as the mediating roles of self-esteem (SE) and social support (SS). Based on social cognitive theory, this study initially constructed a theoretical model to examine the impact of health awareness on healthy lifestyles among university students. Self-esteem and social support were introduced as mediating variables to further develop a chain mediation model. A total of 1169 participants (554 males and 615 females) completed an online questionnaire survey. In this study, participants filled out the Health Awareness Scale (HAS), the Diet and Healthy Lifestyle Scale (DEVS), the Rosenberg Self- esteem Scale (RSES), and the Oslo Social Support Scale (OSSS-3). The results showed that high levels of health awareness positively influenced self-esteem, which in turn positively affected social support, ultimately leading to a positive impact on healthy lifestyles. Specifically, self-esteem and social support played significant mediating roles between health awareness and healthy lifestyles. Overall, the findings reveal the pathways through which health awareness affects healthy lifestyles, highlighting important implications for designing effective interventions to promote healthy lifestyles.

**Keywords:** health awareness; healthy lifestyles; self-esteem; social support

#### 1. Introduction

In the context of increasingly fierce social competition, the pressure of daily life continues to rise, particularly among university students, often leading them to adopt unhealthy lifestyles [1]. They commonly face issues such as unhealthy eating habits, lack of sleep, and sedentary behavior with insufficient physical exercise. This lifestyle negatively impacts their physical and mental health [2]. Previous research has shown that a balanced diet and an active lifestyle play crucial roles in preventing chronic non-communicable diseases. By maintaining a balanced diet and a positive attitude towards life, individuals can effectively reduce the risk of these diseases [3]. Adopting healthy living habits not only enhances the quality of life but also helps alleviate stress in a busy daily schedule [4]. Exploring the healthy lifestyles of university students contributes to improving their overall health levels and reducing the incidence of future health problems.

Health awareness refers to an individual's cognition and attention to their health status and related behaviors. It should not only focus on health behaviors themselves but also emphasize individuals' attitudes toward health. Measured through self-health awareness assessment, personal responsibility, and health motivation, health awareness encompasses not only specific health behaviors but also the psychological state changes these behaviors induce [5]. University students generally lack health awareness; despite being in the prime of their physical health, their attention to disease and health is relatively low. At the same time, the accelerated pace of life, rapid changes in the external environment, and diverse schedules pose many challenges to maintaining healthy lifestyles [6]. Previous studies have shown that individual health awareness significantly influences health behaviors and lifestyles. Enhancing health awareness can not only slow down the progression of lifestyle diseases but also motivate continuous improvement in health status [7]. Therefore, health awareness may be an important predictor of healthy lifestyles among university students. We hypothesize that health awareness positively influences university students' healthy lifestyles. Although previous studies have confirmed the correlation between health awareness and healthy lifestyles, few have explored the underlying mechanisms of this relationship in depth.

Social cognitive theory, originating from learning theory, proposes an interaction model between cognition, behavior, and environment [8]. The theory suggests that an individual's cognition and behavior interact with the environment and emphasizes the important role of cognitive factors in this interaction process [9]. Specifically, social cognitive theory posits that human behavior is influenced not only by the external environment but also driven by internal cognitive processes. An individual's health awareness, an internal cognitive drive, prompts the adoption of healthy lifestyles [7]. This focus on health behavior can enhance an individual's sense of self-esteem. Individuals with high self-esteem are more likely to believe in their ability to engage in healthy behaviors. Whether an individual is in adolescence or adulthood, self-esteem plays a crucial role in overall health [10]. There is a positive correlation between self-esteem and healthy lifestyles; individuals with high levels of self-esteem are more likely to engage in healthy behaviors, such as maintaining

good dietary habits and regular exercise [11]. Therefore, we hypothesize that health awareness indirectly influences healthy lifestyles through the mediating effect of self- esteem.

Social support is a mutual assistance behavior among people, manifested as care, understanding, and recognition. It includes not only emotional support but also practical help. Social support comes in various forms and can be obtained through family members, friends, colleagues, and community organizations [12]. Studies have shown a positive correlation between social support and adolescents' health behaviors. This means that obtaining emotional and practical support from family, friends, or the community helps adolescents actively participate in physical activities and maintain healthy lifestyles. Such support not only increases their willingness to participate in exercise but also provides the necessary resources and motivation, making it easier for them to adhere to regular physical activity [13]. Parental and peer social support significantly influence adolescents' lifestyle behaviors. Specifically, the more support they receive from parents and peers, the more actively they participate in physical activities and increase their intake of fruits and vegetables [14,15]. Enhanced health awareness can improve individuals' interactions within their social support network, thereby promoting the implementation of healthy behaviors [16,17]. Therefore, we hypothesize that health awareness indirectly influences healthy lifestyles through the mediating effect of social support.

Health awareness encompasses a wide range of health knowledge, attitudes, and behaviors [18], including physical health, mental health, nutrition, exercise, and disease prevention. Research has shown that reproductive health awareness can significantly positively predict individuals' self-esteem levels; adolescents with higher reproductive health awareness tend to exhibit higher self-esteem levels [19]. Additionally, self-esteem is positively correlated with healthy lifestyles; individuals with high levels of self-esteem are more likely to adopt healthy lifestyles, such as maintaining a balanced diet, thereby improving their overall health status [20]. When individuals have lower levels of self-esteem, they are more likely to exhibit unhealthy behaviors, such as eating disorders [21]. Furthermore, while there is no significant correlation between self-esteem and parental relationships, there is a strong link between self-esteem and perceived social support. This means that, although parental relationships may have a limited impact on self-esteem, perceived social support from friends, colleagues, or the broader community plays an important role in enhancing self-esteem [22]. Therefore, we hypothesize that health awareness influences healthy lifestyles through the chain mediating effect of self-esteem and social support.

In summary, we have constructed a chain mediation model to explore the impact of health awareness on university students' healthy lifestyles. This model proposes four hypotheses: health awareness has a significant positive impact on university students' healthy lifestyles (H1); self-esteem mediates the relationship between health awareness and healthy lifestyles (H2); social support mediates the relationship between health awareness and healthy lifestyles (H3); and self-esteem and social support sequentially mediate the

relationship between health awareness and healthy lifestyles (H4). Through this model, we can gain a deeper understanding of how health awareness, by enhancing self-esteem and obtaining social support, further promotes university students in adopting positive healthy lifestyles.

#### 2. Material and Methods

2.1. Participants

We used a random sampling method to recruit university students from a university in Guangdong Province, China, for this study. Recruitment started on March 23, 2024, and ended on April 12, 2024. All participants voluntarily participated and submitted their questionnaires through an online survey. A total of 1297 students participated in the survey, and we conducted a rigorous inspection and screening of the collected data based on the following criteria: (1) excluding questionnaires with invalid personal information, such as incomplete or contradictory information; (2) excluding questionnaires with invalid responses, such as those with overly consistent answers where all questions were answered with the same option. Ultimately, 1169 participants (554 males and 615 females) were included in the study. The average age of the participants was 20.56 years (standard deviation = 1.730; range = 18 to 23 years). Before the formal start of the study, we provided each participant with a detailed informed consent form, ensuring they fully understood the purpose, process, and potential impact of the study. Only after obtaining their explicit consent were they included in the research sample.

2.2. Measures
     2.2.1 Health Awareness

This study used the Health Awareness Scale (HAS) [23] to measure the health awareness of university students. The scale consists of 6 items rated on a 7-point Likert scale ranging from 1 (strongly disagree) to 7 (strongly agree). Higher total scores indicate higher levels of health awareness. In this study, the Cronbach’s alpha coefficient for the scale was 0.991.

2.2.2 Healthy Lifestyles

This study used the Diet and Healthy Lifestyle Scale (DEVS) [24] to measure the healthy lifestyles of university students. The scale consists of 14 items, with responses limited to 3 options for each item, ranging from 1 (minimal perceived intake) to 3 (maximum perceived intake). Higher total scores indicate higher levels of healthy lifestyles. In this study, the Cronbach’s alpha coefficient for the scale was 0.932.

2.2.3 Self-esteem

This study used the Rosenberg Self-esteem Scale (RSES) [25], which consists of 10 items rated on a 4-point Likert scale ranging from 1 (strongly disagree) to 4 (strongly agree). Higher total scores indicate higher levels of self-esteem. In this study, the Cronbach’s alpha coefficient for the scale was 0.996.

2.2.4 Social Support

This study used the Oslo Social Support Scale (OSSS-3) [26], which consists of 3 items. The first item is rated on a 4-point Likert scale, and the second and third items are rated on a 5-point Likert scale. Higher total scores indicate higher levels of social support. In this study, the Cronbach’s alpha coefficient for the scale was 0.981.

2.3. Statistical Analyses

This study used the PROCESS macro in SPSS 27.0 to analyze the data. Harman’s single factor test was used to prevent common method bias. Descriptive statistics and correlation analyses were conducted to examine the relationships among health awareness, healthy lifestyles, self-esteem, and social support. To test the significance of the chain mediation model, model 6 in the PROCESS macro was employed [27]. To eliminate the impact of multicollinearity, the significance of the mediation effect was tested using the Bootstrap method with a confidence interval set at 95% and 5000 iterations.

2.4. Ethical Considerations

This study was approved by the Ethics Committee of Guangdong Business and Technology University on February 5, 2024, with the approval number 2024GS006. To ensure the privacy of the participants, this study was conducted anonymously. Before the study began, we provided all participants with a detailed informed consent form and obtained their written consent.

#### 3. Results

3.1. Common Method Deviation Test

The results of Harman's single factor test showed that there were four factors with eigenvalues greater than 1, and the variance explained by the largest factor was 38.106% (less than 40%). Therefore, no significant common method bias was detected.

3.2. Preliminary Analyses

Descriptive statistics and correlation analysis results are shown in Table 1. There were significant positive correlations among health awareness, healthy lifestyles, self-esteem, and social support.

## Table 1

| Means, standard deviations and correlation matrices of variables (*N* =1169). | | | | | | |
| --- | --- | --- | --- | --- | --- | --- |
|  | M | SD | 1 | 2 | 3 | 4 |
| 1.HA | 20.200 | 7.713 | 1 |  |  |  |
| 2.HL | 22.847 | 7.007 | .208*** | 1 |  |  |
| 3.SE | 24.502 | 11.001 | .101** | .384*** | 1 |  |
| 4.SS | 6.824 | 3.813 | .122*** | .405*** | .124*** | 1 |

### Notes: **p* < 0.05, ***p* < 0.01, ****p* < 0.001. HA = Health Awareness; HL= Healthy Lifestyles; SE = Self-esteem; SS = Social Support. The following were the same.

3.3. Chain Mediation Analyses

The results of the chain mediation analysis are shown in Figure 1, and the regression analysis results are presented in Table 2. Health awareness significantly positively predicted healthy lifestyles (β = 0.189, p < 0.001), self-esteem (β = 0.145, p < 0.001), and social support (β = 0.055, p < 0.001). Additionally, self-esteem significantly positively predicted healthy lifestyles (β = 0.208, p < 0.001) and social support (β = 0.039, p < 0.001), and social support significantly positively predicted healthy lifestyles (β = 0.641, p < 0.001). After including self-esteem and social support as mediating variables, the predictive effect of health awareness on healthy lifestyles remained significant (β = 0.120, p < 0.001). These findings indicate that self-esteem and social support partially mediate the relationship between health awareness and healthy lifestyles.

Fig. 1. The result of chain mediation model.

***p < 0.001. The data of all variables were standardized.

## Table 2

**Regression analysis between variables (N =1169).**

| **Dependent variables** | **Predictive variables** | **R** | **R2** | **F** | **β** | **t** | **Boot 95 % CI** |
| --- | --- | --- | --- | --- | --- | --- | --- |
| HL |  | 0.54 | 0.29 | 161.97*** |  |  |  |
|  | HA |  |  |  | 0.12 | 5.32*** | [0.08,0.17] |
|  | SE |  |  |  | 0.21 | 13.14*** | [0.18,0.24] |
|  | SS |  |  |  | 0.64 | 13.97*** | [0.55,0.73] |
| SS |  | 0.17 | 0.03 | 16.44*** |  |  |  |
|  | HA |  |  |  | 0.06 | 3.81*** | [0.03,0.08] |
|  | SE |  |  |  | 0.04 | 3.88*** | [0.02,0.06] |
| SE |  | 0.10 | 0.01 | 12.12*** |  |  |  |
|  | HA |  |  |  | 0.15 | 3.48*** | [0.06,0.23] |

### Notes: *p < 0.05, **p < 0.01, ***p < 0.001. The data of all variables were standardized, the following were the same.

The mediation effect analysis results are shown in Table 3. The mediation effect consists of the indirect effects of three paths. Path 1: HA→SE→HL (β = 0.030, 95% CI [0.013, 0.049]); Path 2: HA→SS→HL (β = 0.035, 95% CI [0.017, 0.055]); Path 3:

HA→SE→SS→HL (β = 0.004, 95% CI [0.001, 0.007]). All three paths, Path 1, Path 2, and Path 3, were significant.

**Table 3. Indirect effect of HA on HL via SE and SS (N =1169).**

| **Effect** | **Product of Coefficients** | | **Boot95% CI** | |
| --- | --- | --- | --- | --- |
|  | **Effect** | **Boot Se** | **LLCI** | **ULCI** |
| HA→ SE →HL | 0.030 | 0.009 | 0.013 | 0.049 |
| HA→SS→HL | 0.035 | 0.010 | 0.017 | 0.055 |
| HA→ SE →SS→HL | 0.004 | 0.002 | 0.001 | 0.007 |
| Direct effects: HA→HL | 0.120 | 0.023 | 0.076 | 0.165 |
| Total indirect effect | 0.069 | 0.014 | 0.041 | 0.097 |
| Total effect | 0.189 | 0.026 | 0.138 | 0.240 |

### Notes: HA = Health Awareness; HL= Healthy Lifestyles; SE = Self-esteem; SS = Social Support.

#### 4. Discussion

In this study, we constructed a chain mediation model among university students to explore the relationship between health awareness and healthy lifestyles, as well as the mediating roles of self-esteem and social support. The results showed a significant positive correlation between health awareness and healthy lifestyles, with health awareness significantly positively predicting healthy lifestyles (H1). Further analysis revealed that self- esteem mediated the relationship between health awareness and healthy lifestyles (H2), and similarly, social support also mediated this relationship (H3). This study further demonstrated the chain mediation effect of self-esteem and social support in the relationship between health awareness and healthy lifestyles (H4).

Firstly, the results of this study indicated a significant positive correlation between health awareness and healthy lifestyles among the participants. The stronger the health awareness of university students, the more likely they are to engage in positive health behaviors. A previous study on the healthy lifestyles of university students indicated that those with higher levels of health awareness are more likely to adopt healthy lifestyles. Compared to non-medical students, medical students have higher cognition, understanding, and perception of health information, which promotes their adoption of healthy lifestyles [28]. Previous research has shown that consumers who are aware of the health benefits of regularly consuming organic products are more willing to pay higher prices for these products. This suggests that health awareness not only prompts people to choose healthier diets but also influences their consumption behaviors [29]. This study's framework further extends this theory, showing that health awareness can also significantly predict other aspects of healthy lifestyles. Based on the results of this study, it can be concluded that enhancing the health awareness of university students can directly promote their adoption of healthier lifestyles, thereby improving overall health levels.

Secondly, the study found that self-esteem mediated the relationship between health awareness and healthy lifestyles. Previous research has shown that individuals with high oral health behavior and self-esteem are closely related to oral health awareness [30]. These studies highlight the importance of health awareness in enhancing self-esteem, further supporting the results of this study. Self-esteem is an individual's evaluation and

perception of themselves and is an important component of self-cognition [31]. High self- esteem means that individuals have a highly positive overall evaluation of themselves, while low self-esteem means that individuals have a more negative evaluation of themselves. Individuals with high levels of self-esteem have better overall health compared to those with low self-esteem levels [32]. Self-esteem is one of the strongest predictors of eating disorders. People with eating disorders typically have low levels of self-esteem. Individuals with low self-esteem are more likely to engage in binge eating and unhealthy dieting behaviors, further illustrating the important role of self-esteem in healthy lifestyles [33]. Self-esteem is positively correlated with positive health behaviors. Previous studies have also demonstrated the importance of self-esteem in understanding and promoting health behaviors [34]. These studies consistently show that individuals with high self- esteem are more inclined to adopt and maintain healthy lifestyles.

Furthermore, social support also plays an important mediating role in the impact of health awareness on healthy lifestyles. Parental and peer social support significantly influences adolescents' lifestyle behaviors, with greater social support making individuals more likely to engage in physical activity and consume fruits and vegetables [35]. Additionally, this support, such as encouragement, role modeling, and sharing healthy goal behaviors, has been shown to significantly improve individuals' adherence to healthy eating and exercise [36]. Enhancing individuals' cognitive beliefs in their ability to make healthy choices and engage in healthy behaviors can reduce the difficulty of execution and ultimately promote the occurrence of healthy behaviors [37]. In other words, improving health awareness can increase individuals' confidence in adopting healthy lifestyles, making it easier to engage in healthy behaviors. Moreover, individuals with high self-esteem typically perceive themselves as having more social support, which is closely associated with various health outcomes, including longevity [38]. Therefore, by enhancing university students' health awareness, their self-esteem and sense of social support can be increased, thereby improving their healthy lifestyles.

This study proposed a chain mediation model to explore the process and mechanisms by which the independent variable (health awareness) influences the dependent variable (healthy lifestyles) by examining the roles of two mediating variables (self-esteem and social support). The results showed that health awareness indirectly promoted university students' healthy lifestyles by enhancing self-esteem and increasing social support. The final sample of this study included 1169 participants, which effectively reduced errors due to a small sample size and increased the reliability of the research findings.

However, this study has some limitations. First, it did not collect variables such as family background and self-efficacy. Research has shown that self-efficacy is an important predictor of healthy lifestyles [39]. Studying the relationship between health awareness and healthy lifestyles while controlling for these personal factors would provide deeper and more comprehensive insights. Second, as the respondents in this study were university students, the results may not be generalizable to other populations. However, studying the healthy lifestyles of young people is particularly important because the habits formed

during university and adolescence may have a profound impact on their lifelong health practices [40]. Finally, this study used a cross-sectional design, so future research should adopt a longitudinal approach to explore changes in health awareness, self-esteem, social support, and healthy lifestyles among university students to gain a more comprehensive understanding of the causal relationships between variables.

Despite these limitations, this study has significant practical and theoretical implications. Theoretically, this study constructed a chain mediation model that reveals how health awareness influences university students' healthy lifestyles through self-esteem and social support, enriching the theoretical foundation in the relevant field and providing a reference framework for future research. Practically, the findings of this study offer valuable insights for enhancing the healthy lifestyles of university students, providing important references for educational institutions and policymakers in developing health education and support policies. Specifically, universities can offer mandatory physical education courses to help students improve self-cognition and health behaviors [41]; additionally, schools, communities, and related departments should strengthen support for university students' health by providing more health resources and social support [42]. These measures can effectively improve university students' healthy lifestyles and promote their overall health levels.

In summary, this study reveals the potential pathways through which health awareness influences the healthy lifestyles of university students. The results indicate a significant positive relationship between health awareness and healthy lifestyles, with health awareness significantly predicting the development of healthy lifestyles. Additionally, health awareness indirectly influences healthy lifestyles through the chain mediation effect of self- esteem and social support. These findings provide important empirical support for developing interventions to enhance the healthy lifestyles of university students.

#### References

[1] Thwaite TL, Heidke P, Williams SL, Vandelanotte C, Rebar AL, Khalesi S. Barriers to healthy lifestyle behaviors in Australian nursing students: A qualitative study. Nursing & Health Sciences 2020;22:921–8. https://doi.org/10.1111/nhs.12749.
[2] Al-Qahtani AM. Lifestyle habits among Najran University students, Najran, Saudi Arabia.	Frontiers	in	Public	Health	2022;10:938062. https://doi.org/10.3389/fpubh.2022.938062.
[3] Alzamil HA, Alhakbany MA, Alfadda NA, Almusallam SM, Al-Hazzaa HM. A profile of physical activity, sedentary behaviors, sleep, and dietary habits of Saudi college female students. Journal of Family and Community Medicine 2019;26:1–8. https://doi.org/10.4103/jfcm.JFCM_58_18.
[4] Shetty S, Kamath N, Nalini M. Academic Stress and Study Habits of Health Science University Students. Journal of Health and Allied Sciences NU 2022;12:71–5. https://doi.org/10.1055/s-0041-1730746.
[5] Hong H. Scale development for measuring health consciousness: Re- conceptualization. That Matters to the Practice 2009:212.
[6] Mollborn S, Lawrence EM, Saint Onge JM. Contributions and Challenges in Health Lifestyles Research. J Health Soc Behav 2021;62:388–403. https://doi.org/10.1177/0022146521997813.
[7] Nagai M. Relationships among lifestyle awareness, age, and lifestyle-related diseases in healthy Japanese community residents. Asian/Pacific Island Nursing Journal 2020;5:103.
[8] Bandura A. Social cognitive theory of moral thought and action. Handbook of moral behavior and development, Vol. 1: Theory; Vol. 2: Research; Vol. 3: Application, Hillsdale, NJ, US: Lawrence Erlbaum Associates, Inc; 1991, p. 45–103.
[9] Bandura A. Self-efficacy: the exercise of control. 12. print. New York: Freeman; 1997.
[10] Jafflin K, Pfeiffer C, Bergman MM. Effects of self-esteem and stress on self-assessed health: a Swiss study from adolescence to early adulthood. Quality of Life Research 2019;28:915–24. https://doi.org/10.1007/s11136-018-2059-1.
[11] Sampasa-Kanyinga H, Lien A, Hamilton HA, Chaput J-P. Canadian 24-h Movement guidelines, life stress, and self-esteem among adolescents. Frontiers in Public Health 2022;10:702162. https://doi.org/10.3389/fpubh.2022.702162.
[12] RL K. Conboys over the life course: Attachment roles and social support. Life-Span Development and Behavior 1980;3:253–86.
[13] Spink KS, Shields CA, Chad K, Odnokon P, Muhajarine N, Humbert L. Correlates of structured and unstructured activity among sufficiently active youth and adolescents: A new approach to understanding physical activity. Pediatric Exercise Science 2006;18:203–15. https://doi.org/10.1123/pes.18.2.203.
[14] Pugliese J, Tinsley B. Parental socialization of child and adolescent physical activity: a meta-analysis. Journal of Family Psychology 2007;21:331. https://doi.org/10.1037/0893-3200.21.3.331.
[15] Villard LC, Rydén L, Ståhle A. Predictors of healthy behaviours in Swedish school children. European Journal of Preventive Cardiology 2007;14:366–72.
[16] Kelly ME, Duff H, Kelly S, McHugh Power JE, Brennan S, Lawlor BA, et al. The impact of social activities, social networks, social support and social relationships on the cognitive functioning of healthy older adults: a systematic review. Systematic Reviews 2017;6:1–18. https://doi.org/10.1186/s13643-017-0632-2.
[17] Li F, Luo S, Mu W, Li Y, Ye L, Zheng X, et al. Effects of sources of social support and resilience on the mental health of different age groups during the COVID-19 pandemic. BMC Psychiatry 2021;21:1–14.
[18] Johnson EJ, Hariharan S. Public health awareness: knowledge, attitude and behaviour of the general public on health risks during the H1N1 influenza pandemic. Journal of Public Health 2017;25:333–7. https://doi.org/10.1007/s10389-017-0790-7.
[19] Mohammadi S, Rabiee N, Motaghi Z. Study of the relationship between awareness of reproductive health and self-esteem level in the students of Shahroud University of Medical Sciences. International Journal of Health Studies 2019.
[20] Hazzard VM, Telke SE, Simone M, Anderson LM, Larson NI, Neumark-Sztainer D. Intuitive eating longitudinally predicts better psychological health and lower use of disordered eating behaviors: findings from EAT 2010–2018. Eating and Weight Disorders-Studies on Anorexia, Bulimia and Obesity 2021;26:287–94.
[21] Cella S, Cipriano A, Aprea C, Cotrufo P. Self-Esteem and binge eating among adolescent boys and girls: The role of body disinvestment. International Journal of Environmental Research and Public Health 2021;18:7496. https://doi.org/10.3390/ijerph18147496.
[22] Safwa K, Shari B. Relationship with parents, Perceived social support and Self-esteem among Young adults. International Journal of Indian Psychȯlogy 2022;10.
[23] Michaelidou N, Hassan LM. The role of health consciousness, food safety concern and ethical identity on attitudes and intentions towards organic food. International Journal of Consumer Studies 2008;32:163–70. https://doi.org/10.1111/j.1470- 6431.2007.00619.x.
[24] Calizaya-Milla YE, Saintila J, Morales-García WC, Ruiz Mamani PG, Huancahuire- Vega S. Evidence of validity and factorial invariance of a diet and healthy lifestyle scale (DEVS) in university students. Sustainability 2022;14:12273. https://doi.org/10.3390/su141912273.
[25] Rosenberg M. Society and the Adolescent Self-Image. Princeton University Press; 1965. https://doi.org/10.1515/9781400876136.
[26] Kocalevent R-D, Berg L, Beutel ME, Hinz A, Zenger M, Härter M, et al. Social support in the general population: standardization of the Oslo social support scale (OSSS-3). BMC Psychology 2018;6:1–8. https://doi.org/10.1186/s40359-018-0249-9.
[27] Hayes AF. PROCESS: A versatile computational tool for observed variable mediation, moderation, and conditional process modeling. University of Kansas, KS; 2012.
[28] Yang S-C, Luo Y-F, Chiang C-H. The associations among individual factors, eHealth literacy, and health-promoting lifestyles among college students. Journal of Medical Internet Research 2017;19:e15. https://doi.org/10.2196/jmir.5964.
[29] Tan BC, Chai LT, Min PS. Attitude towards eating “green”: Do consumer consciousness, healthy lifestyle, and value orientation matter. Conference Paper, 2017, p. 153–60.
[30] Lee S-L, Kwag J-S, Choi J-H. Correlation and influencing factors on oral health awareness, oral health behavior, self-esteem and OHIP-14 in childcare teachers. Journal of Korean Society of Dental Hygiene 2015;15:261–9.
[31] Baumeister RF, Campbell JD, Krueger JI, Vohs KD. Does high self-esteem cause better performance, interpersonal success, happiness, or healthier lifestyles? Psychological Science in the Public Interest 2003;4:1–44. https://doi.org/10.1111/1529-1006.01431.
[32] Glendinning A. Family life, health and lifestyles in rural areas: the role of self‐esteem. Health Education 1998;98:59–68. https://doi.org/10.1108/09654289810199829.
[33] French SA, Leffert N, Story M, Neumark-Sztainer D, Hannan P, Benson PL. Adolescent binge/purge and weight loss behaviors: Associations with developmental assets. Journal of Adolescent Health 2001;28:211–21. https://doi.org/10.1016/S1054- 139X(00)00166-X.
[34] Ma X. Health outcomes of elementary school students in New Brunswick: The education	perspective.	Evaluation	Review	2000;24:435–56. https://doi.org/10.1177/0193841X0002400501.
[35] Sallis JF, Prochaska JJ, Taylor WC. A review of correlates of physical activity of

children and adolescents. Medicine and Science in Sports and Exercise 2000;32:963–

75. https://doi.org/10.1097/00005768-200005000-00014.

[36] Barrera Jr M, Toobert DJ, Angell KL, Glasgow RE, MacKinnon DP. Social support and social-ecological resources as mediators of lifestyle intervention effects for type 2 diabetes. Journal	of	Health	Psychology	2006;11:483–95. https://doi.org/10.1177/1359105306063321.
[37] Kelly SA, Melnyk BM, Jacobson DL, O’Haver JA. Correlates among healthy lifestyle cognitive beliefs, healthy lifestyle choices, social support, and healthy behaviors in adolescents: implications for behavioral change strategies and future research. Journal	of	Pediatric	Health	Care	2011;25:216–23. https://doi.org/10.1016/j.pedhc.2010.03.002.
[38] Ornish D. Love and survival: The scientific basis for the healing power of intimacy. Harper Collins; 2011.
[39] Lo SWS, Chair SY, Lee FK. Factors associated with health-promoting behavior of people with or at high risk of metabolic syndrome: based on the health belief model. Applied	Nursing	Research	2015;28:197–201. https://doi.org/10.1016/j.apnr.2014.11.001.
[40] Huntsinger ET, Luecken LJ. Attachment relationships and health behavior: The mediational role of self-esteem. Psychology & Health 2004;19:515–26. https://doi.org/10.1080/0887044042000196728.
[41] Konczos C, Bognár J, Szakály Z, Barthalos I, Simon I, Oláh Z. Health awareness, motor performance and physical activity of female university students. Biomedical Human Kinetics 2012;4:12–7. https://doi.org/10.2478/v10101-012-0003-3.
[42] Hanawi SA, Saat NZM, Zulkafly M, Hazlenah H, Taibukahn NH, Yoganathan D, et al. Impact of a Healthy Lifestyle on the Psychological Well-being of University Students. International Journal of Pharmaceutical Research & Allied Sciences 2020;9.

Figure	[Click here to access/download;Figure;Figure 1.png](https://www2.cloud.editorialmanager.com/pone/download.aspx?id=36841541&guid=d8eaa45b-60cb-4425-9686-58ca8f79d071&scheme=1)[<https://www2.cloud.editorialmanager.com/pone/download.aspx?id=36841541&guid=d8eaa45b-60cb-4425-9686-58ca8f79d071&scheme=1>](https://www2.cloud.editorialmanager.com/pone/download.aspx?id=36841541&guid=d8eaa45b-60cb-4425-9686-58ca8f79d071&scheme=1)

Data Review URL

[Click here to download Data Review URL http://doi.org/10.3886/E207741V1](http://doi.org/10.3886/E207741V1)
