# Supplementary material for: The influence of health awareness on university students’ healthy lifestyles: The chain mediating role of self-esteem and social support
Source: PLoS One. 2024 Oct 10;19(10):e0311886. doi: 10.1371/journal.pone.0311886 (PMC11466429; doi:10.1371/journal.pone.0311886)
Supplement: Attachment — Submitted filename: 20240917-Response to Reviewers.docx [file pone.0311886.s003.docx]

**Rebuttal Letter**

Dear Academic Editor and Reviewers,

Thank you for reviewing our manuscript, "The Influence of Health Awareness on University Students' Healthy Lifestyles: The Chain Mediating Role of Self-esteem and Social Support." We have carefully considered all the feedback provided and have made the necessary revisions to address each point. Below is a detailed response to the suggestions provided:

Reviewer 1:

Clarify Research Objective: We have explicitly stated the research objective at the beginning of the paper to provide readers with a clearer understanding of the study's purpose and direction.

Problem Statement: We have clarified the problem statement in the introduction, explaining that the hypothesis was developed based on theoretical foundations and a gap identified in the literature.

Mediator Role: We have provided a detailed explanation of the mediator's role and its function within the context of this research in the literature review section.

Sample Selection Process: We have elaborated on the sample selection process in the "Participants" section, including details on how the random sampling method was implemented.

Online Survey Process and Limitations: We have detailed the process by which participants voluntarily participated and submitted their questionnaires through an online survey, and discussed any limitations associated with using an online survey, along with the measures taken to mitigate them.

Instrument Translation: We have added a description of the translation process of the instruments and how the translated instruments were applied to the sample.

Literature Review Section: As recommended, we have created a separate literature review section instead of including it in the introduction.

Discussion, Limitations, and Future Research: We have thoroughly explained and clearly outlined these sections in the revised manuscript.

Reviewer 2:

Abstract: We have added 1-2 sentences briefing on the study before stating the aims. Additionally, we have been more specific about the theoretical and practical implications.

Introduction: We have relocated each hypothesis to the related literature review section.

Participants Section: In the "Materials and Methods" section, we have elaborated on how the random sampling method was implemented.

Measures Section: We have described the back-to-back translation process involved in adopting the questionnaires and included 1-2 examples of items for each measure.

Results Section: We have added a descriptive analysis of the demographic profiling of participants in the Results section.

Common Method Deviation Test Section: We have included the necessary citations for the Common Method Deviation Test.

Journal Requirements:

Compliance with PLOS ONE Style Requirements: We have ensured that the manuscript meets PLOS ONE's style requirements, including those for file naming.

Data Availability Statement: We confirm that our manuscript contains all the raw data required to replicate the results of our study.

Reference List Review: We have carefully reviewed the reference list to ensure that it is complete and correct. We confirm that there are no citations of retracted papers, and therefore, no deletions or replacements were necessary. All references cited in the list are current and valid.

We hope these revisions have adequately addressed all your suggestions and have improved the overall quality of the manuscript. Thank you for your time and effort in reviewing our work.

Sincerely,

Zeqing Zhang
